# Intracellular Drug Delivery Process of Am80-Encapsulated Lipid Nanoparticles Aiming for Alveolar Regeneration

**DOI:** 10.3390/ph16060838

**Published:** 2023-06-04

**Authors:** Tomomi Akita, Kazuaki Oda, Satoru Narukawa, Yuki Morita, Kota Tange, Yuta Nakai, Chikamasa Yamashita

**Affiliations:** 1 Department of Pharmaceutics and Drug Delivery, Faculty of Pharmaceutical Sciences, Tokyo University of Science, 2641 Yamazaki, Noda 278-8510, Japan; 3b20517@ed.tus.ac.jp (K.O.); 3b22553@ed.tus.ac.jp (S.N.); 3b19559@ed.tus.ac.jp (Y.M.); 2 Life Science Research Laboratory, NOF CORPORATION, 3-3 Chidori-cho, Kawasaki-ku, Kawasaki City 210-0865, Japan; kota_tange@nof.co.jp (K.T.); yuta_nakai@nof.co.jp (Y.N.)

**Keywords:** chronic obstructive pulmonary disease, Am80 (tamibarotene), alveolar regeneration, lipid nanoparticle, pulmonary administration

## Abstract

Chronic obstructive pulmonary disease (COPD) results in obstructive ventilatory impairment caused by emphysema, and current treatment is limited to symptomatic therapy or lung transplantation. Therefore, the development of new treatments to repair alveolar destruction is especially urgent. Our previous study revealed that 1.0 mg/kg of synthetic retinoid Am80 had a repair effect on collapsed alveoli in a mouse model of elastase-induced emphysema. From these results, however, the clinical dose calculated in accordance with FDA guidance is estimated to be 5.0 mg/60 kg, and it is desirable to further reduce the dose to allow the formulation of a powder inhaler for clinical application. To efficiently deliver Am80 to the retinoic acid receptor in the cell nucleus, which is the site of action, we focused on SS-cleavable proton-activated lipid-like material O-Phentyl-P4C2COATSOME^®^SS-OP, hereinafter referred to as “SS-OP”). In this study, we investigated the cellular uptake and intracellular drug delivery process of Am80-encapsulated SS-OP nanoparticles to elucidate the mechanism of Am80 by nanoparticulation. Am80-encapsulated SS-OP nanoparticles were taken up into the cells via ApoE, and then Am80 was efficiently delivered into the nucleus via RARα. These results indicated the usefulness of SS-OP nanoparticles as drug delivery system carriers of Am80 for COPD treatment.

## 1. Introduction

Chronic obstructive pulmonary disease (COPD) is an intractable respiratory disease caused by chronic inhalation exposure to toxic substances, mainly cigarette smoke, that results in permanent airflow obstruction due to bronchial obstruction and alveolar destruction [1]. COPD affects approximately 300 million people worldwide and is the third leading cause of death worldwide [2]. The risk of severe disease in COVID-19, which is currently prevalent worldwide, is also associated with chronic respiratory diseases, including COPD [3]. However, COPD is becoming a global disease. There are currently no drugs that can repair alveolar destruction during the disease course [4]. Symptomatic treatment includes long-acting muscarinic antagonists, inhaled steroids, and long-acting β2 agonists [5], but satisfaction with COPD treatment remains at 60% [6,7]. Therefore, the development of new treatments to repair alveolar destruction is especially urgent.

With the increasing importance of regenerative medicine in recent years for the repair of injured tissues, promoting lung regeneration is a promising therapeutic strategy for COPD [6]. Studies of endogenous stem/progenitor cells and differentiation-inducing agents have attracted attention as a means to regenerate and treat damaged lungs [8]. The existence of tissue progenitor cells in the lung was also suggested, and human alveolar epithelial progenitor cells were identified [9]. The alveoli are specialized for gas exchange as terminal structures of the distal airways, and their surfaces are occupied by type I and type II alveolar epithelial cells [10]. In COPD, the irreversible destruction of alveoli makes gas exchange difficult, and it is considered that a complete cure of COPD can be achieved by the differentiation of alveolar epithelial progenitor cells into type I or type II alveolar epithelial cells that constitute the alveoli and regenerating the alveoli.

The vitamin A derivative retinoic acid (all-trans retinoic acid; ATRA) has been reported to be an important signaling molecule for differentiation [11,12,13]. ATRA is an active metabolite of vitamin A and has been used clinically to treat acute promyelocytic leukemia because of its high differentiation potential. Retinoic acid has also been found to play an important role in the development and maturation repair of alveoli in the respiratory tract [14]. However, a clinical study of the oral administration of ATRA in patients with COPD did not show a significant therapeutic effect, suggesting that ATRA may have been metabolized by CYP26A1 [15,16]. Therefore, we focused on tamibarotene (4-[(5, 6, 7, 8-thetrahydro-5, 5, 8, 8-tetramethyl-2 naphthyl) carbamoyl] benzoic acid (Am80, Figure S1A), a synthetic retinoid. Am80 was developed to improve the therapeutic effect and reduce the side effects of ATRA [17]. It is an agonist of the retinoic acid receptor (RAR)-like ATRA and has higher receptor selectivity for RARα and β than ATRA [17]. Our laboratory has reported that Am80 exerts alveolar repair effects in a mouse model of COPD after pulmonary administration at a dose of 1.0 mg/kg [18]. From these results, the clinical dose calculated in accordance with FDA guidance [19] is estimated to be 5.0 mg/60 kg, and thus, it is desirable to further reduce the dose to formulate a powder inhaler for clinical application.

Since the site of action of Am80 is the nuclear receptor RAR [20,21], we thought that the nuclear concentration of Am80 would need to be increased to reduce the dose of Am80. For this purpose, efficient drug delivery into the cytoplasm is first considered necessary. Therefore, we focused on SS-cleavable proton-activated lipid-like material SS-OP (Figure S1B), which was developed as a nucleic acid delivery carrier [22]. SS-OP has two intracellular environmental response units: “tertiary amines” and “disulfide bonds” [22]. After internalization in endosomes, SS-OP nanoparticles exhibit a proton-sponge effect in which the surface of the particle is positively charged, allowing it to disrupt and escape the endosome via protonation of a tertiary amine in response to a decrease in pH associated with endosomal maturation [22]. Furthermore, the surface is destabilized by the cleavage of the intramolecular disulfide bond by glutathione in the cytoplasm; the drug is released via self-disintegration through the intraparticle reaction between the resulting thiol group and the intramolecular phenyl ester, such that efficient drug delivery to the cytoplasm can be expected [22]. Recently, we reported that Am80-encapsulated SS-OP nanoparticles showed a therapeutic effect in a mouse model of COPD at a dose that was 1/100 that of Am80 [23]. In this previous report, we were able to show pharmacological effects at different doses in the free Am80 and Am80-encapsulated SS-OP nanoparticles, but it was unclear whether there were differences in the nuclear translocation of Am80. In addition, since the mechanism by which encapsulation of Am80 in nanoparticles showed efficacy at a dose of 1/100 has not been clarified, it is necessary to investigate the properties of SS-OP nanoparticles in vitro.

In this study, we investigated the usefulness of SS-OP nanoparticles as drug delivery system (DDS) carriers in the efficient intranuclear delivery of Am80 for the radical treatment of COPD by clarifying the intracellular drug delivery process of SS-OP nanoparticles encapsulated with Am80.

## 2. Results and Discussion

### 2.1. Cellular Drug Delivery Process of SS-OP Nanoparticles in the Intracellular Transport of Am80

#### 2.1.1. Characterization of Am80-Encapsulated SS-OP Nanoparticles

In this study, we investigated the intracellular uptake and intracellular drug delivery process of SS-OP nanoparticles encapsulated with Am80. We prepared Am80-encapsulated SS-OP nanoparticles and evaluated their physical properties. Particle size, zeta potential, and Am80 encapsulation efficiency were measured as physical properties of the prepared Am80-encapsulated SS-OP nanoparticles solution, and the results are shown in Table 1. The particle size distribution of the nanoparticles followed a uniform unimodal distribution. In general, nanoparticles with a positive charge on the surface are more likely to be taken up by cells. On the other hand, in vivo, they tend to aggregate due to electrostatic interactions with biological components [24]. Therefore, the charge on the surface of the nanoparticles used in this study is near neutral to avoid interactions with biological components. In addition, setting the charge on the surface to neutral makes it easier for nanoparticles to aggregate, so DMG-PEG2000 is added to prevent it.

#### 2.1.2. Intracellular Uptake of SS-OP Nanoparticles via ApoE

**Figure 1:**
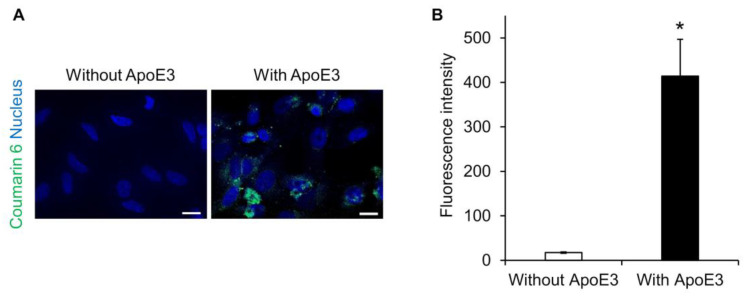
SS-OP is dependent on ApoE for cellular uptake. (**A**) Nuclear staining of HeLa cells with DAPI and treatment with SS-OP nanoparticles labeled with coumarin 6, which was observed with a BZ9000 fluorescence microscope, ×20. Scale bar, 20 µm. The concentration of Am80 used for particle preparation was 5 mM (2 μmol), and Coumarin 6 was used at 0.02 μmol, which is 0.5% of the total lipid content, at a concentration of 1 mM. The SS-OP solution was diluted 100 times and used for the treatment. (**B**) The fluorescence intensity of coumarin 6. Data represent the mean ± S.E. (*n* = 3). * *p* < 0.05, Welch’s *t*-test.

Lipid nanoparticles are known to adsorb ApoE on their surfaces and to be taken up into hepatocytes via a low-density lipoprotein receptor [25,26,27]. Therefore, we investigated whether ApoE is also involved in the intracellular uptake of SS-OP nanoparticles. SS-OP nanoparticles encapsulating coumarin 6 as a fluorescent dye were mixed with ApoE and exposed for 30 min to HeLa cells not expressing ApoE. Fluorescence microscopy observation revealed that the intracellular uptake of SS-OP nanoparticles was enhanced by the ApoE, as the fluorescence values of coumarin 6 were significantly increased in the ApoE-supplemented group, whereas coumarin 6 fluorescence was almost absent in the non-supplemented group (Figure 1). This suggested that SS-OP nanoparticles are taken up into cells by ApoE-mediated endocytosis similar to general lipid nanoparticles. ApoE is an important plasma lipoprotein involved in cholesterol transport and metabolism. Unlike other apolipoproteins, which are synthesized mainly in the liver, ApoE is synthesized in the liver, brain, spleen, lung, skin, kidney, and ovary [28]. In addition, increased production of ApoE in the lungs has been reported in inflammatory diseases of the lungs [29,30,31]. Therefore, we investigated the expression level of ApoE in the lung of a mouse model of elastase-induced COPD. The results showed that the production of ApoE increased similarly in the COPD model mice (Figure S2). Although ApoE was originally present in the lung, it was further increased with COPD modeling, so it is expected that ApoE is involved in the uptake of SS-OP nanoparticles into lung cells in the COPD model as well.

**Figure 2:**
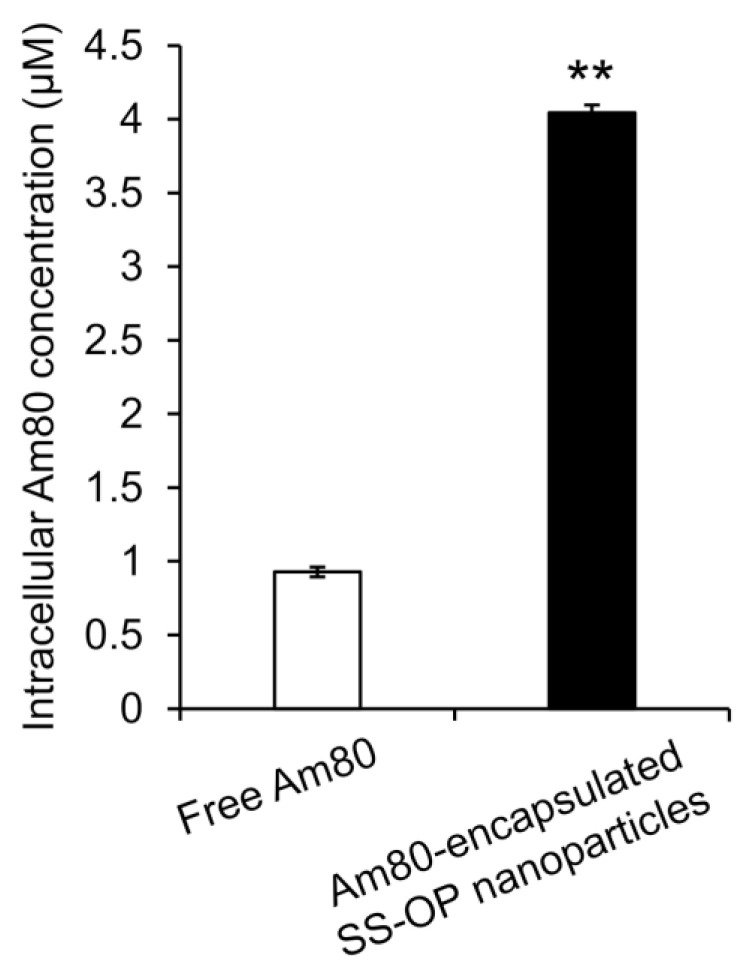
Intracellular Am80 concentration by treatment with free Am80 or Am80-encapsulated SS-OP nanoparticles under ApoE exposure in Calu-6 cells. Each datum represents the mean ± S.E. (*n* = 3). ** *p* < 0.01, Student *t*-test.

To investigate whether Am80 was efficiently delivered into cells by SS-OP nanoparticles compared with Am80 alone, Am80-encapsulated SS-OP nanoparticles mixed with ApoE and Am80 alone were exposed to Calu-6 cells for 30 min. As a result, the amount of Am80 in the cells significantly increased in the group treated with Am80-encapsulated SS-OP nanoparticles compared with the group treated with Am80 alone (Figure 2), and SS-OP nanoparticles were found to efficiently deliver Am80 into cells. The above suggested that Am80 could be efficiently delivered into alveolar epithelial cells via ApoE in elastase-induced COPD model mice by pulmonary administration of Am80-encapsulated SS-OP nanoparticles.

#### 2.1.3. Endosomal Escape Ability of SS-OP Nanoparticles

**Figure 3:**
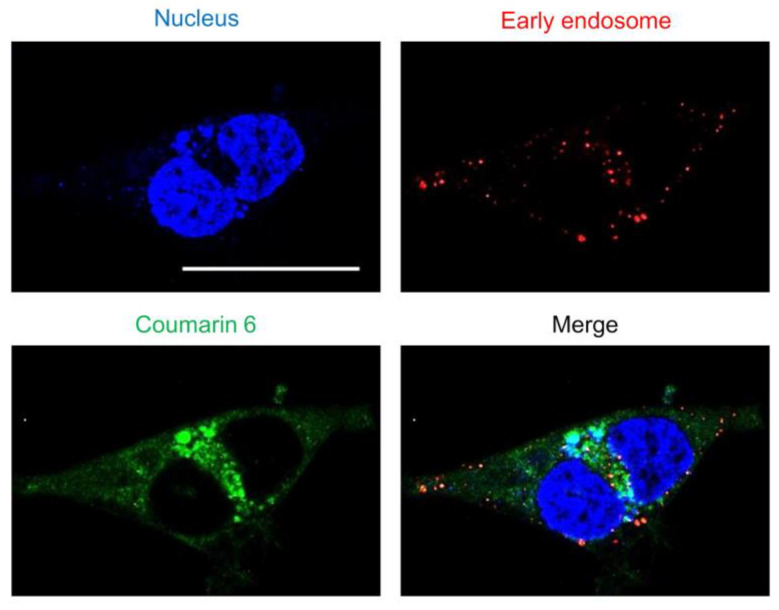
The endosomal escape of SS-OP nanoparticles. Calu-6 cells stained for early endosomes with RFP and the nucleus with Hoechst 33342, trihydrochloride, trihydrate, and treated with SS-OP nanoparticles labeled with coumarin 6 were observed with a confocal microscope, ×63, water immersion. Scale bar: 30 µm.

Substances are taken up by endocytosis either fused with lysosomes and were metabolized or escaped from endosomes and translocated to the cytoplasm [32]. Nanoparticles adsorbed on cells are taken up by endocytosis, after which endosomes activate a proton pump that gradually lowers the internal pH [33]. As the endosome then fuses with a lysosome and the contents are degraded by enzymes, it is important for the nanoparticles to escape from the endosome as quickly as possible. To evaluate the endosomal escape ability of the “tertiary amine” characteristic of SS-OP nanoparticles, early endosomes were stained. As observed via confocal laser scanning microscopy, coumarin-6-labeled SS-OP nanoparticles were slightly localized to early endosomes, but most were observed to diffuse into the cytoplasm (Figure 3). This indicated that SS-OP nanoparticles escape from endosomes and migrate into the cytoplasm efficiently after internalization. In addition to this result, the increase in intracellular uptake of SS-OP nanoparticles by ApoE in Figure 1 is considered to be the reason why the intracellular amount of Am80 increased compared to the free form in Figure 2.

#### 2.1.4. Drug Release by Cleavage of Disulfide Bonds in SS-OP Nanoparticles

**Figure 4:**
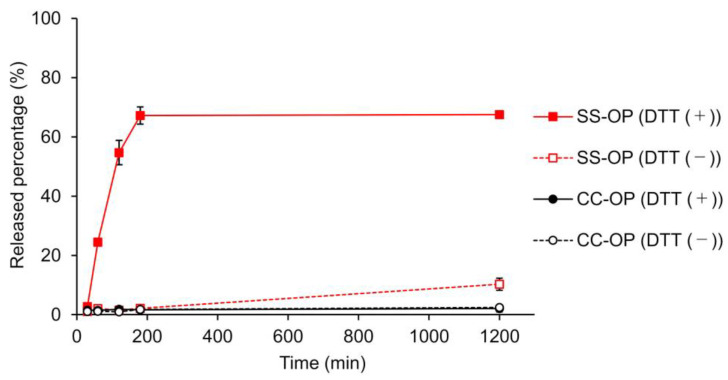
Calcein leakage from SS-OP or CC-OP nanoparticles when 10 mM DTT was added with incubation at 37 ℃. Released percentage (%) was calculated by the fluorescence intensity of each sample divided by that of Triton X-treated sample. The solid lines indicate the groups of incubation with 10 mM DDT (DTT (+)), and the dashed lines indicate the groups of incubation without DTT (DTT (−)). The red lines indicate SS-OP, and the black lines indicate CC-OP. Each datum represents the mean ± S.E. (n = 3).

The release of the drug from the nanoparticle is important to obtain an immediate effect of the encapsulated drug after the nanoparticle enters the cytoplasm. To this end, carriers incorporating disulfide bonds that cleave in the cytoplasmic-reducing environment have been designed, and SS-OP has also been designed for the disintegration of particle and drug release by disulfide bonds and subsequent intraparticle reactions with phenyl esters [22,34]. To investigate whether SS-OP nanoparticles release drugs by cleavage of disulfide bonds, SS-OP nanoparticles and CC-OP nanoparticles were incubated under 10 mM DTT as a reducing agent, and the drug release rate was calculated. CC-OP nanoparticles have carbon chains substituted for disulfide bonds in SS-OP. As a result, CC-OP showed little drug release with or without the reducing agent. In contrast, SS-OP did not release many drugs under physiological conditions, but drug release occurred rapidly under reducing conditions, and drug release of approximately 70% was observed 180 min after nanoparticle exposure (Figure 4). The SS-OP disulfide bond is reversibly cleaved by cytoplasmic glutathione reduction [22]. The thiolate of glutathione reacts with a disulfide bond. Since DTT is also a reducing agent with thiolate, it is thought that the disulfide bond is cleaved via the reduction reaction. These results suggest that the disulfide bonds of SS-OP nanoparticles play an important role in realizing the rapid release of drugs from nanoparticles under the reducing environment in the cytoplasm.

### 2.2. Intranuclear Deliverability of Am80 Using SS-OP Nanoparticles

#### 2.2.1. Nuclear Transport of Am80 by RARα

Retinoic acid regulates gene expression by activating several transcription factors of the nuclear receptor family, including RAR. Retinoic acid transport proteins are present in the cytoplasm for the nuclear transport of retinoic acid and are regulated by retinoic acid binding. It was recently reported that RAR is localized in the cytoplasm, and RAR translocates to the nucleus and activates transcription of target genes upon retinoic acid binding [35,36]. As Am80 is a kind of retinoid, it is expected to bind to the retinoic acid transport protein and be carried to the nucleus. Additionally, Am80 is a selective agonist of RAR and has an especially high affinity with RARα rather than with other subtypes. We focused on RARα, an Am80 binding protein, as a mechanism for promoting the nuclear translocation of Am80 released into cytoplasm from SS-OP nanoparticles.

**Figure 5:**
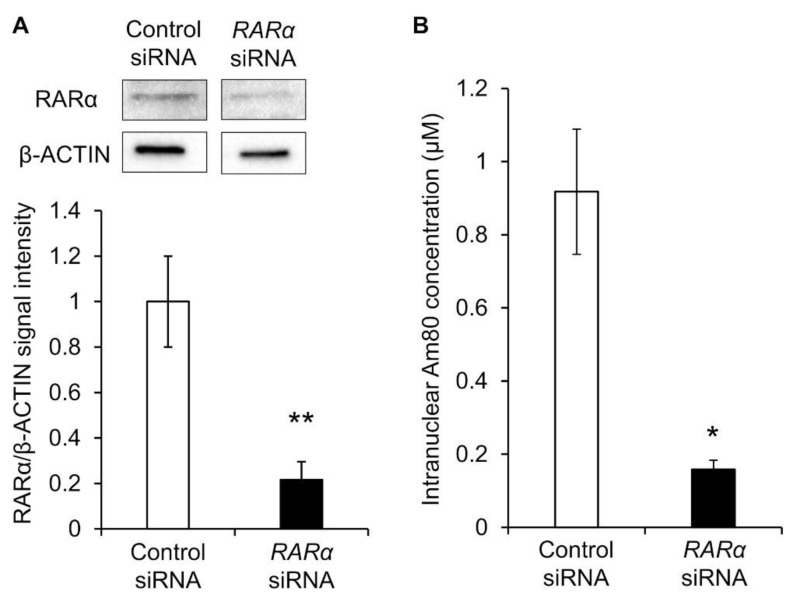
The nuclear translocation of Am80. (**A**) Representative Western blot images and the relative expression of RARα in Calu-6 measured after siRNA knockdown. The data were normalized using β-actin as a housekeeping gene. Each datum represents the mean (*n* = 3) ± S.E. ** *p* < 0.01, Student *t*-test. (**B**) Intranuclear Am80 concentration following treatment with Am80-encapsulated SS-OP nanoparticles in Calu-6 with RARα knockdown. Each datum represents the mean ± S.E. (*n* = 3). * *p* < 0.05, Welch’s *t*-test.

Therefore, RARα was knocked down, and the nuclear translocation amount of Am80 was measured. siRNA RARα was exposed to Calu-6 cells, and its knockdown efficiency was evaluated. β-actin and RARα proteins were detected via Western blotting, and the reference ratio was calculated using β-actin as the housekeeping gene. Compared with the control siRNA group, *RARα* siRNA caused a knockdown of approximately 80% (Figure 5A). Calu-6 cells knocked down by RARα were exposed to Am80-encapsulated SS-OP nanoparticles for 30 min. As a result, the nuclear translocation was significantly decreased in the RARα knockdown group compared with the control group. This suggested that RARα was involved in the nuclear translocation of Am80 (Figure 5B).

#### 2.2.2. Intranuclear Deliverability of Am80 by SS-OP Nanoparticles

**Figure 6:**
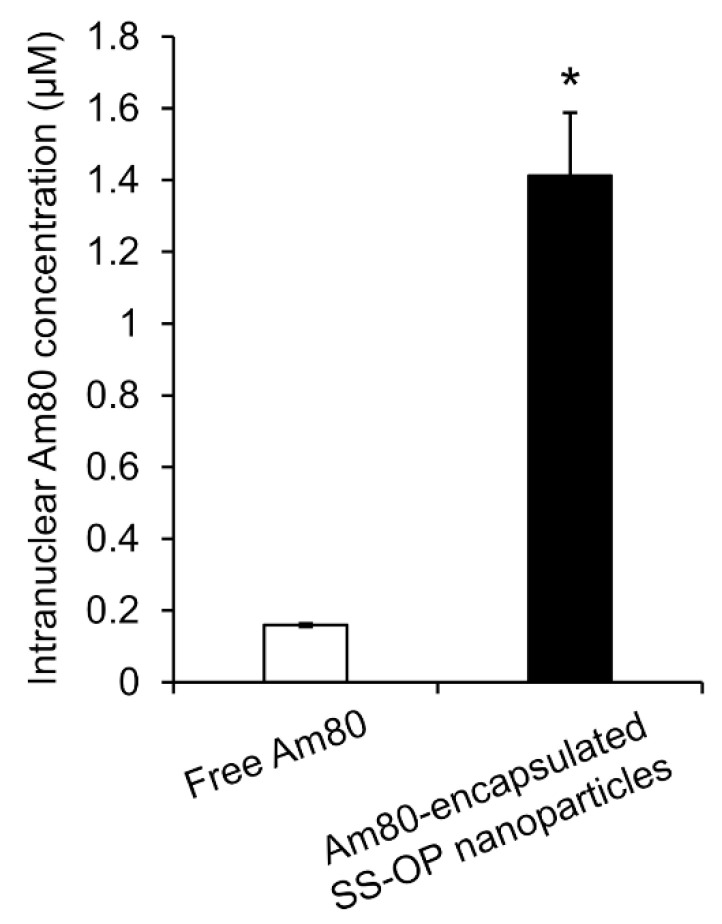
Intranuclear Am80 concentration by treatment with free Am80 or Am80-encapsulated SS-OP nanoparticles under ApoE exposure in Calu-6 cells. Each datum represents the mean ± S.E. (*n* = 3). * *p* <0.05, Welch’s *t*-test.

To investigate whether Am80 was efficiently delivered to the cell nucleus as the site of action via SS-OP nanoparticles compared with Am80 alone, Am80-encapsulated SS-OP nanoparticles mixed with ApoE and Am80 alone were exposed to Calu-6 cells for 30 min. The amount of Am80 in the nucleus significantly increased in the group treated with Am80-encapsulated SS-OP nanoparticles compared with the group treated with free Am80 alone (Figure 6). Am80 was found to be more efficiently delivered to the nucleus using SS-OP nanoparticles.

**Figure 7:**
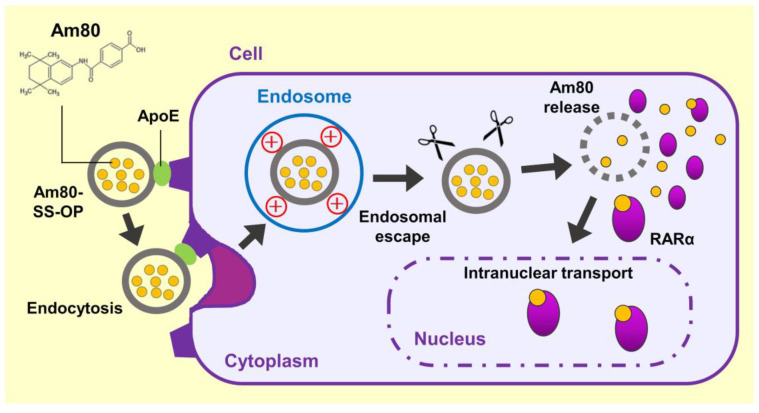
Scheme of the efficient intranuclear transport mechanism of Am80 by SS-OP nanoparticles.

These findings suggested that Am80-encapsulated SS-OP nanoparticles were taken up into cells via ApoE, and the Am80 was released into the cytoplasm, where it bound to RARα and was delivered into the cell nucleus, the site of action (Figure 7). (1) The incorporation of SS-OP nanoparticles into cells is promoted by ApoE. ApoE increases in the lung of a mouse model of elastase-induced COPD, and ApoE is involved in intracellular uptake. (2) SS-OP nanoparticles taken up by endocytosis escape from the endosomes and migrate into the cytoplasm by the proton sponge effect of the tertiary amine of the SS-OP nanoparticles. Tertiary amines protonated by endosomes with acidic pH induce an extensive inflow of ions and water into the endosomes. Then, the increased osmotic pressure subsequently leads to the rupture of the endosomal membrane. (3) SS-OP nanoparticles disintegrate and release Am80 by cleavage of disulfide bonds under a reducing environment of glutathione in the cytoplasm. The thiolate of glutathione reacts with the disulfide bond and cleavages it. The nucleophilic attack of the thiolate of SS-OP undergoes the intramolecular phenyl ester, leading to a reaction that produces the final products of degradation. (4) Am80 released from SS-OP nanoparticles bound to RARα and delivered into the cell nucleus.

## 3. Materials and Methods

### 3.1. Preparation of Am80-Encapsulated SS-OP Nanoparticles

Am80 was a gift from Dr. Koichi Shudo at the University of Tokyo (Tokyo, Japan). SS-OP, COATSOME^®^ ME-8181 (DOPE), and SUNBRIGHT^®^ GM-20 (DMG-PEG2000) were provided by NOF CORPORATION (Tokyo, Japan). Cholesterol was provided by Tokyo Chemical Industry Co., Ltd. (Tokyo, Japan).

Am80-encapsulated SS-OP nanoparticles were prepared via the antisolvent dilution method. First, 2.0 µmol Am80, 1.2 µmol SS-OP, 1.6 µmol DOPE, 1.2 µmol cholesterol, and 0.4 µmol DMG-PEG2000 were dissolved in 1.6 mL ethanol (FUJIFILM Wako Pure Chemical Corporation [FUJIFILM Wako], Osaka, Japan). Malic acid buffer was prepared with DL-Malic Acid (Nacalai Tesque, Kyoto, Japan) and sodium chloride (NaCl; FUJIFILM Wako). Particles were precipitated by adding 1.6 mL of malic acid buffer (20 mM [pH 3.0], 1250 mM NaCl) to the lipid–ethanol solution. Subsequently, the solvent was replaced with saline (Otsuka Pharmaceutical Co., Ltd. Tokyo, Japan) by ultrafiltration.

The particle size and zeta potential of the Am80-encapsulated SS-OP nanoparticles were measured using a Zetasizer (ELSZ-2000; Otsuka Electronics Co., Ltd., Osaka, Japan), and the encapsulation rate of the Am80 was determined via high-performance liquid chromatography (HPLC) (Nexera X2; Shimadzu Corporation, Kyoto, Japan).

### 3.2. Animals

Male 5-week-old ICR mice were purchased from Sankyo Labo Service Corporation (Tokyo, Japan). Animals were housed in a temperature-controlled (23 ± 1 °C) facility maintained on a 12 h light/dark cycle with standard food available ad libitum. This experiment was conducted with the approval of the Tokyo University of Science Ethics Committee (approval number: Y21031; date of approval: 30 April 2021).

### 3.3. Preparation of Elastase-Induced-COPD Model Mice

We previously reported the pulmonary administration method used [37]. Mice were anesthetized with isoflurane (MSD Animal Health K.K, Osaka, Japan) and held in a Mouse Intubation Platform-Model MIP (Penn-Century, Wyndmoor, PA, USA). Subsequently, the airway was located using a Small Animal Laryngoscope (Model LS-2; Penn-Century) as a tracheal endoscope for mice, and a stainless-steel oral sonde (N-PK002; Nihon Bioresearch Inc., Gifu, Japan) was introduced into the airway. The drug solution was administered in synchronization with the air intake of the mouse.

The COPD model mice were generated via pulmonary administration of 50 μL saline, in which 4.5 U porcine pancreatic elastase (Elastin Products Company, Inc., Owensville, MO, USA) was dissolved, to 6-week-old male ICR mice.

### 3.4. Measurement of ApoE Concentration in BALF

After intraperitoneal injection of a 100-μL pentobarbital sodium (70 mg/kg)-xylazine (12 mg/kg) anesthesia mixture, mice with loss of righting reflex were immediately killed by exsanguination. Then, the bronchus was incised, and bronchoalveolar lavage fluid (BALF) was collected with 1 mL of saline.

Mouse Apolipoprotein E Simple Step ELISA^®^ Kit (ab215086; Abcam PLC, Cambridge, UK) was used for assay according to the manufacturer’s protocol. The BALF sample was diluted and used as a sample for measurement. All reagents were used after being brought to room temperature. The diluted sample or standard solution and the specific antibody solution were added to each well and shaken at room temperature for 1 h. Each well was washed with wash buffer. Then, a light-emission detection reagent was added, and the solutions were shaken for 10 min in a light-shielding condition. Finally, reaction stop solution was added to each well, and after shaking for 1 h, the absorbance at 450 nm was measured using an Envision system (Perkin Elmer Japan G.K., Kanagawa, Japan), and the apolipoprotein E (ApoE) concentration was calculated.

### 3.5. Cell Culture

Calu-6 cells (ATCC, Manassas, VA, USA) and HeLa cells (JCRB9004; established by Gey et al. and obtained from the Japanese Collection of Research Bioresources [JCRB] Cell Bank, National Institutes of Biomedical Innovation, Health and Nutrition) were cultured in E-MEM (FUJIFILM Wako) supplemented with 10% fetal bovine serum (Life Technology Co., New York, NY, USA), MEM non-essential amino acids solution (FUJIFILM Wako), 1 mM sodium pyruvate solution (FUJIFILM Wako), and 0.1% penicillin streptomycin (Life Technology Co., New York, NY, USA) in a humidified 5% CO_2_ atmosphere maintained at 37 °C.

### 3.6. Evaluation of Intracellular Uptake Pathways

Coumarin-6-labeled SS-OP nanoparticles were prepared by adding 20 nmol of coumarin 6 (Tokyo Chemical Industry Co., Ltd.) to a lipid–ethanol solution. Human recombinant ApoE3 (1 μg/mL; FUJIFILM Wako) was mixed with coumarin-6-labeled SS-OP nanoparticles at 37 °C for 1 h. Then, 1 × 10^4^ HeLa cells were seeded on 8-well culture slides and incubated at 37 °C overnight. Coumarin-6-labeled nanoparticle solution (200 µL; diluted with 10% E-MEM to a lipid concentration of 55 µM) was placed in each well and left in the incubator at 37 °C for 30 min. Cells were washed with PBS, fixed at room temperature with 4% paraformaldehyde for 15 min, and washed again with PBS. DAPI (4’,6-diamidino-2-phenylindole; Roche Applied Sciences Co., Mannheim, Germany) solution (100 μL/well) was added to the wells and reacted at room temperature for 90 min. After the reaction, cells were washed with PBS-T and observed with a BZ-9000 fluorescence microscope (KEYENCE CORPORATION, Osaka, Japan), and fluorescence intensity was measured with Image J analysis software (National Institutes of Health, Bethesda, MD, USA).

### 3.7. Observation of Intracellular Drug Delivery Process and Measurement of Drug Release

CellLight^TM^ Early Endosomes-RFP, BacMam 2.0 (Thermo Fisher Scientific Inc., Tokyo, Japan) was used for early endosomal staining. Calu-6 cells were seeded into a 35 mm glass base dish at 1 × 10^4^ cells/dish and incubated at 37 °C overnight. Coumarin-6-labeled nanoparticles were mixed with Hoechst 33342, trihydrochloride, and trihydrate (Thermo Fisher Scientific Inc.), which was diluted with 10% E-MEM to 2.4 μg/mL. The 200 μL of mixed coumarin-6-labeled nanoparticle and Hoechst solution was placed in each well and left in the incubator at 37 °C for 30 min. After washing with PBS, fluorescence observation was performed with a TCS SP8 confocal microscope (Leica, Wetzlar, Germany), and image analysis was performed with Leica Application Suite X Software (Leica).

To investigate the effect of the presence or absence of disulfide bonds on drug release of nanoparticles, SS-OP and CC-OP nanoparticles were fluorescently labeled with calcein (Tokyo Chemical Industry Co., Ltd.). CC-OP (ccPalmO-Phe) is a lipid in which disulfide bonds in the SS-OP structure were replaced by carbon chains. Calcein-labeled nanoparticles were prepared using dissolved malic acid buffer with 2 mM calcein solution. Calcein-labeled nanoparticles were diluted with saline to 1 mM and dithiothreitol (DTT) (FUJIFILM Wako) to 10 mM. They were incubated at 37 °C for 30, 60, 120, 180, and 1200 min. Each reaction solution was ultrafiltrated at 14,000× *g*, and the filtrate was subjected to fluorescence measurement using the Envision system at an absorption wavelength of 490 nm and a fluorescence wavelength of 520 nm. Released percentage (%) was calculated by the fluorescence intensity of each sample divided by that of Triton X-100 (FUJIFILM Wako) -treated sample.

### 3.8. Determination of Am 80 Intracellular and Nuclear Translocations by Am 80-Encapsulated SS-OP Nanoparticles

Calu-6 cells were seeded at 5 × 10^6^ cells/dish and incubated at 37 °C for 1 day, followed by exposure to 50 μM of Am80 or Am80-encapsulated SS-OP nanoparticles diluted in E-MEM medium for 30 min. The cells were trypsinized and recovered in E-MEM. Collected cells were centrifuged at 1000 rpm for 5 min to precipitate them, and after the medium was removed, they were resuspended in 200 μL PBS to serve as cell samples.

Intracellular Am80 was extracted by the following intracellular liquid extraction method. Cell samples were centrifuged at 1000× *g* for 10 min, and 150 μL DMSO (Fujifilm Wako) was added to the precipitate. After sonication for 10 min and centrifugation at 20,000× *g* for 5 min, the supernatant was used as a measurement sample. Quantification was performed by HPLC.

Nuclear Am80 was extracted using the following nuclear fractionation extraction method. Cell samples were centrifuged at 1000× *g* for 10 min, and the precipitate was resuspended with 100 μL Buffer-1 (10 mM Tris-HCl [pH 7.5], 1.5 mM EDTA, and 10% glycerol) and allowed to cool on ice for 10 min. The mixture was then centrifuged at 750× *g* for 10 min. The precipitate was resuspended with 100 μL Buffer-2 (10 mM Tris-HCl [pH 7.5] and 5 mM MgCl_2_) and centrifuged at 750× *g* for 10 min. Then, 150 μL DMSO was added to the precipitate, which was sonicated for 10 min and centrifuged at 20,000× *g* for 5 min, following which the supernatant was used as a measurement sample. Quantification was performed by HPLC.

### 3.9. Knockdown by siRNA RARα

Silencer Select Validated siRNA RARα (Thermo Fisher Scientific Inc., Tokyo, Japan) was diluted with nuclease-free water to 10 μM. Then, 7.5 μL Lipofectamine^®^ RNAiMAX Transfection Reagent (Thermo Fisher Scientific Inc.), 3 μL siRNA, and 125 μL Opti-MEM^®^ I (Life Technology Co., New York, NY, USA) were mixed and incubated for 5 min. Then, the Calu-6 was added at a ratio of 10% and allowed to react for 48 h.

### 3.10. Western Blotting

Cultured Calu-6 cells were collected with cold sampling buffer (0.5 M Tris-HCl [pH 6.8], 10% SDS, and Milli-Q water) after being washed with cold PBS. The resulting cell suspensions were quantified by a BCA protein assay using a Pierce^®^ BSATM Protein Assay Kit (Thermo Fisher Scientific Inc.). The sample was diluted with PBS to 200 μg/mL, mixed in equal volumes with sample buffer (0.5 M Tris-HCl [pH 6.8], 10% SDS, Milli-Q water, glycine [FUJIFILM Wako], 1 mg/mL BPB, and 2-ME), and then warmed at 95 °C for 3 min.

First, the protein of interest was separated via sodium dodecyl sulfate-polyacrylamide gel electrophoresis (SDS-PAGE) and transferred to a membrane (Immobilon-P PVDF membrane, Merck, USA). TBS-T (0.1% Tween 20 in 0.05 M Tris-HCl, 0.138 M NaCl, and 0.0027 M KCl [FUJIFILM Wako]) was prepared, and non-specific protein binding was blocked using blocking buffer (0.3% *w/v* nonfat dry milk in 0.1% Tween 20 containing TBS-T) at room temperature for 1 h. Each membrane was then immersed in a primary antibody solution diluted with blocking buffer. They were allowed to permeate overnight at 4 °C. After being washed with TBS-T, the membranes were immersed in secondary antibody solutions diluted with blocking buffer and were shaken at room temperature for 1 h. After the reaction, they were washed again with TBS-T and reacted with Immobilon^TM^ Western Chemiluminescent HRP Substrate at room temperature for 5 min, after which the bands were detected with ChemiDoc MP (Bio-Rad, Hercules, CA, USA).

In this study, anti-RARα (diluted 1:1500, ab28767, Abcam PLC) and rabbit anti-actin antibody (diluted 1:10,000, bs-0061R; Bioss, Woburn, MA, USA) were used as primary antibodies, and donkey anti-goat IgG (HRP) (diluted 1:2000, sc-2020; Santa Cruz Biotechnology, Santa Cruz, Inc., CA, USA) and goat anti-rabbit IgG H & L (HRP) (diluted 1:20,000, ab205718; Abcam PLC) were used as secondary antibodies, respectively.

### 3.11. Statistics

Data are expressed as the mean ± standard error (S.E.) or standard deviation (S.D.). The Student *t*-test or Welch’s *t*-test was used to evaluate differences between two groups. A *p* value < 0.05 was considered statistically significant.

## 4. Conclusions

This study clarified that Am80 could be efficiently delivered into the nucleus by RARα after Am80-encapsulated SS-OP nanoparticles were taken up into cells via ApoE and through endosomal escape and drug release from the nanoparticles. SS-OP nanoparticles are potentially useful as DDS carriers for efficient intranuclear delivery of Am80. These results indicate that efficient cytoplasmic delivery of drugs by SS-OP nanoparticles could also be applied to respiratory diseases. Since the use of SS-OP nanoparticles increases the intracellular uptake of drugs, it is possible to reduce the dose of drugs, and compounds that have not shown efficacy so far may be able to attract attention again as drug candidates. As the next research, we plan to develop a dry powder inhalation of Am80-encapsulated SS-OP nanoparticles. In the future, we would like to find effective drugs not only for COPD but also for other respiratory diseases and evaluate the usefulness of SS-OP nanoparticles.

## Figures and Tables

**Table 1:** Physicochemical properties of Am80-encapsulated SS-OP nanoparticles.

Particle	Am80 Concentration (mM)	Am80 Encapsulated Percent (%)	Particle Size (nm)	P.I.	Zeta Potential (mV)
SS-OP (Am80)	1.86 ± 0.21	74.7 ± 8.31	138 ± 2.85	0.068 ± 0.01	−6.43 ± 2.87

P.I., polydispersity index. Each datum represents the mean ± S.D. (n = 3).

## Data Availability

Data is contained within the article are available on request from the corresponding author.

## Supplementary Materials

The following supporting information can be downloaded at: https://www.mdpi.com/article/10.3390/ph16060838/s1, Figure S1: Chemical structures. Figure S2: Concentration of ApoE in mouse.
