# Supplementary figures and images for: Intracellular Drug Delivery Process of Am80-Encapsulated Lipid Nanoparticles Aiming for Alveolar Regeneration
Source: Pharmaceuticals (Basel). 2023 Jun 4;16(6):838. doi: 10.3390/ph16060838 (PMC10304494; doi:10.3390/ph16060838)

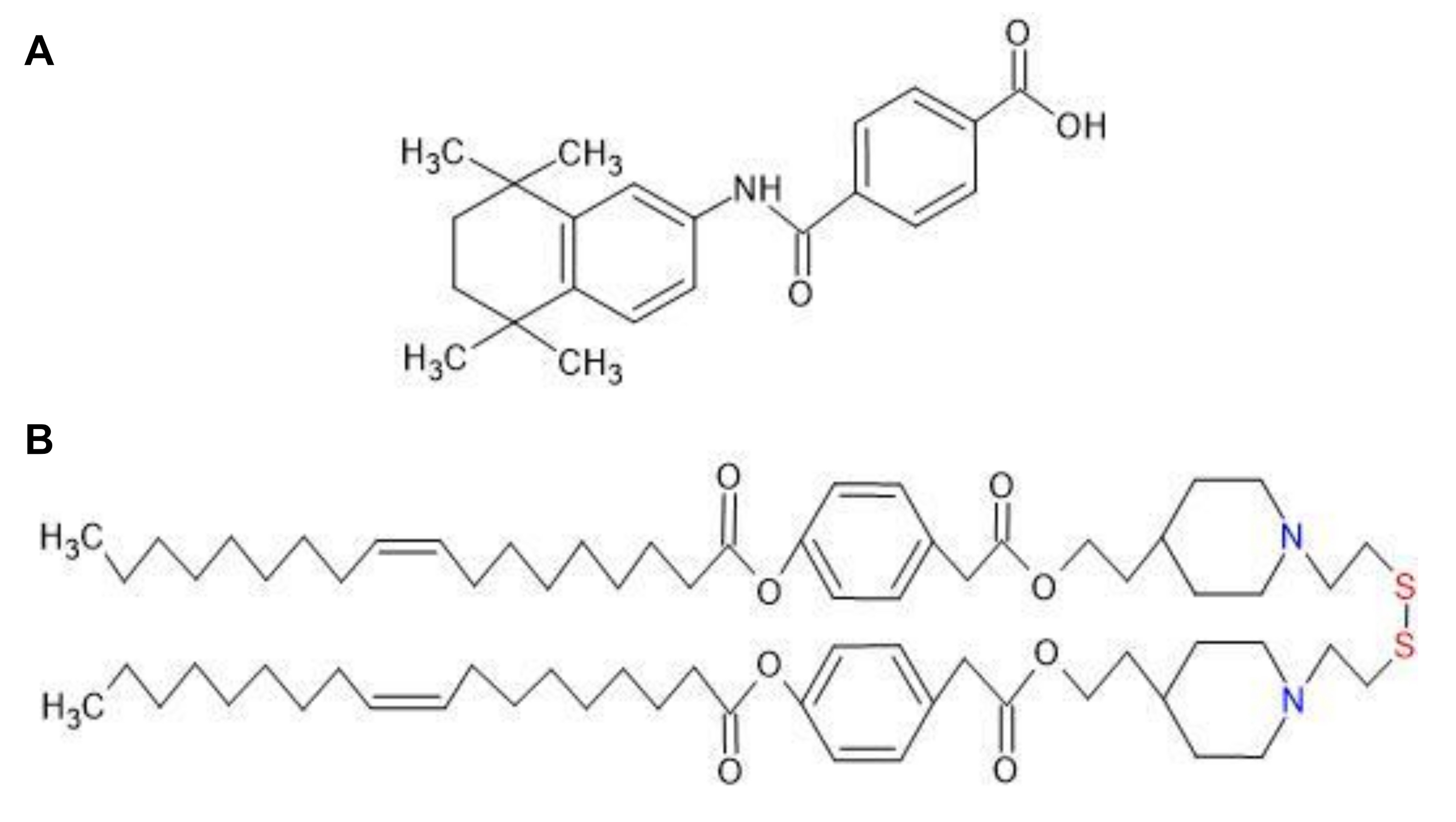

Supplement: Supplementary file 1 [file pharmaceuticals-16-00838-s001.zip › Figure S1.jpg]

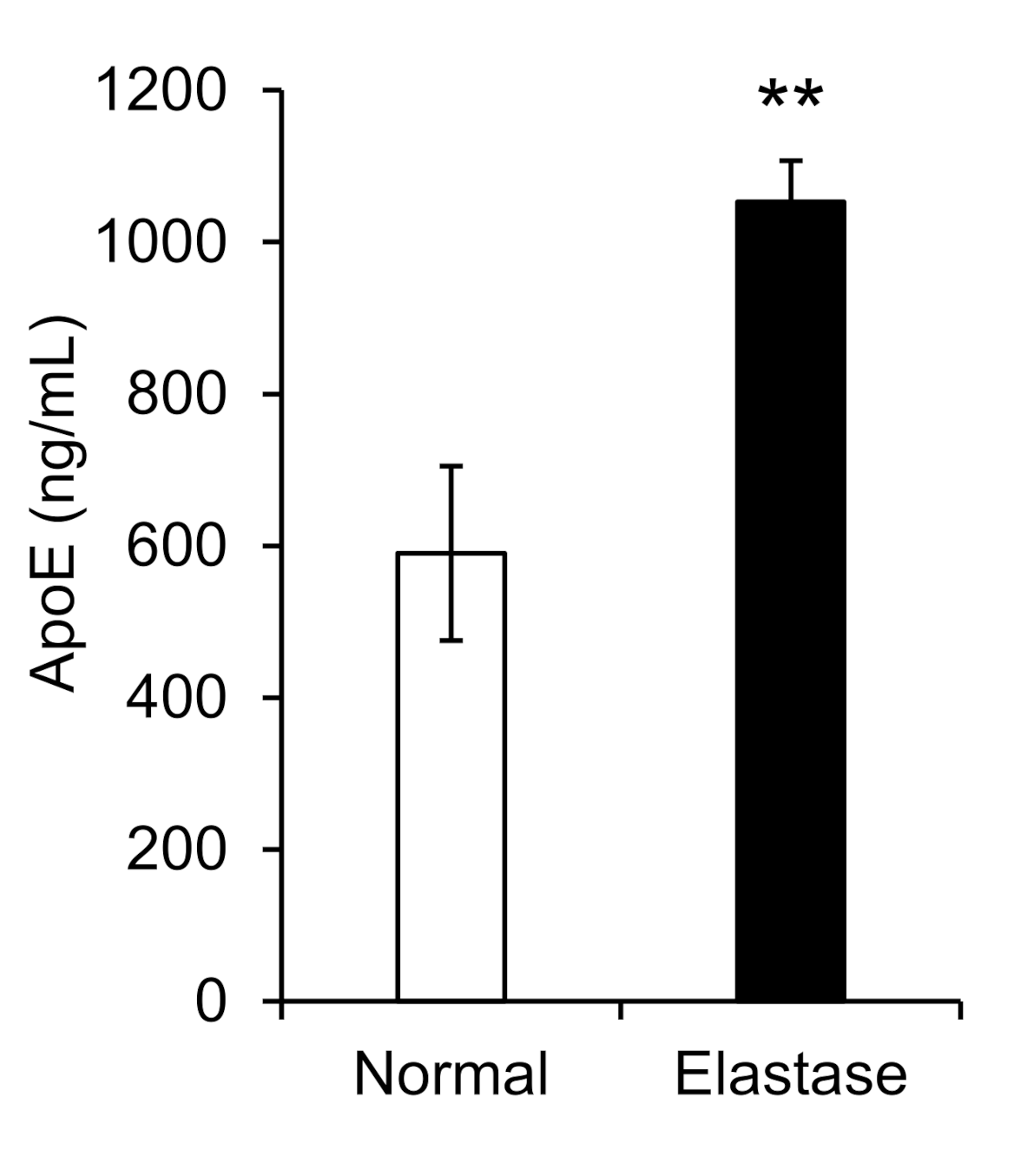

Supplement: Supplementary file 1 [file pharmaceuticals-16-00838-s001.zip › Figure S2.tif]
